# Fibrinogen Gamma Chain Mutations Provoke Fibrinogen and Apolipoprotein B Plasma Deficiency and Liver Storage

**DOI:** 10.3390/ijms18122717

**Published:** 2017-12-15

**Authors:** Francesco Callea, Isabella Giovannoni, Sinan Sari, Esendagli Guldal, Buket Dalgic, Gulen Akyol, Tsuyoshi Sogo, Abdulrahman Al-Hussaini, Giuseppe Maggiore, Andrea Bartuli, Renata Boldrini, Paola Francalanci, Emanuele Bellacchio

**Affiliations:** 1 Department Pathology and Molecular Histopathology, Bambino Gesù Children’s Hospital, IRCCS, 00165 Rome, Italy; isabella.giovannoni@opbg.net (I.G.); renata.boldrini@opbg.net (R.B.); paola.francalanci@opbg.net (P.F.); 2 Department Pediatric Gastroenterology, Gazi University Ankara, 06560 Ankara, Turkey; drsinansari@gmail.com (S.S.); buketdalgic@gmail.com (B.D.); 3 Department Pathology, Gazi University Ankara, 06560 Ankara, Turkey; drguldal@yahoo.com (E.G.); gulenakyol@yahoo.com (G.A.); 4 Department of Pediatric Hepatology and Gastroenterology, Saiseikai Yokohama City Tobu Hospital 3-6-1, Shimosueyoshi, Tsurumi Ward, Yokohama City, Kanagawa, Japan; so5244ibukick@kdp.biglobe.ne.jp; 5 Division of Pediatric Gastroenterology, Children's Specialized Hospital, King Fahad Medical City, College of Medicine, Alfaisal University Riyadh 11525, Saudi Arabia; aa_alhussaini@yahoo.com; 6 Section of Pediatrics, Department of Medical Sciences, University of Ferrara, University Hospital Arcispedale Sant’Anna, 44100 Ferrara, Italy; giuseppe.maggiore@unife.it; 7 Rare Disease and Medical Genetics, Bambino Gesù Children’s Hospital, IRCCS, 00165 Rome, Italy; andrea.bartuli@opbg.net; 8 Genetics and Rare Diseases, Research Division, Bambino Gesù Children’s Hospital, IRCCS, 00165 Rome, Italy; emanuele.bellacchio@opbg.net

**Keywords:** Fibrinogen Storage Disease, APOB-lipoprotein, Endoplasmic Reticulum Storage Disease

## Abstract

p.R375W (Fibrinogen Aguadilla) is one out of seven identified mutations (Brescia, Aguadilla, Angers, Al du Pont, Pisa, Beograd, and Ankara) causing hepatic storage of the mutant fibrinogen γ. The Aguadilla mutation has been reported in children from the Caribbean, Europe, Japan, Saudi Arabia, Turkey, and China. All reported children presented with a variable degree of histologically proven chronic liver disease and low plasma fibrinogen levels. In addition, one Japanese and one Turkish child had concomitant hypo-APOB-lipoproteinemia of unknown origin. We report here on an additional child from Turkey with hypofibrinogenemia due to the Aguadilla mutation, massive hepatic storage of the mutant protein, and severe hypo-APOB-lipoproteinemia. The liver biopsy of the patient was studied by light microscopy, electron microscopy (EM), and immunohistochemistry. The investigation included the DNA sequencing of the three *fibrinogen* and *APOB–lipoprotein regulatory* genes and the analysis of the encoded protein structures. Six additional Fibrinogen Storage Disease (FSD) patients with either the Aguadilla, Ankara, or Brescia mutations were investigated with the same methodology. A molecular analysis revealed the fibrinogen gamma p.R375W mutation (Aguadilla) but no changes in the *APOB* and *MTTP* genes. *APOB* and *MTTP* genes showed no abnormalities in the other study cases. Light microscopy and EM studies of liver tissue samples from the child led to the demonstration of the simultaneous accumulation of both fibrinogen and APOB in the same inclusions. Interestingly enough, APOB-containing lipid droplets were entrapped within the fibrinogen inclusions in the hepatocytic Endoplasmic Reticulum (ER). Similar histological, immunohistochemical, EM, and molecular genetics findings were found in the other six FSD cases associated with the Aguadilla, as well as with the Ankara and Brescia mutations. The simultaneous retention of fibrinogen and APOB-lipoproteins in FSD can be detected in routinely stained histological sections. The analysis of protein structures unraveled the pathomorphogenesis of this unexpected phenomenon. Fibrinogen gamma chain mutations provoke conformational changes in the region of the globular domain involved in the “end-to-end” interaction, thus impairing the D-dimer formation. Each monomeric fibrinogen gamma chain is left with an abnormal exposure of hydrophobic patches that become available for interactions with APOB and lipids, causing their intracellular retention and impairment of export as a secondary unavoidable phenomenon.

## 1. Introduction

Hereditary Hypofibrinogenemia (HH) with hepatic storage of fibrinogen was identified as an Endoplasmic Reticulum Storage Disease (ERSD) in 1987 in an Italian patient [1]. Initially, the entity was accepted with skepticism by fibrinogen experts not only because of its unusual clinical presentation, i.e., hypofibrinogenemia in the absence of overt dysfibrinogenemia, coagulation, or thrombosis manifestations, but also because of its unexpected pathogenesis, i.e., plasma deficiency secondary to a defective export and consequential storage [2]. The final proof that HH with hepatic storage represented a new ERSD was obtained by demonstrating that the retention phenomenon observed in the original Italian patient [1] was due to the p.G284R mutation in the fibrinogen γ chain [3].

The discovery of the new entity revealed the etiology of a disease, fibrinogen storage disease (FSD) [4], that otherwise would have remained cryptogenic, in analogy to the α-1-antitrypsin (AAT) deficiency (AATD) [2].

Since then, six fibrinogen mutant variants were described: Aguadilla p.R375W [5], Angers p.del372-376 [6], Al DuPont p.T314P [7], Pisa p.D316N [8], Beograd p.G366S [8], and Ankara p.H340D [9].

All variants were detected by morphological examination of liver tissue specimens obtained for unexplained liver disease. All reported cases displayed cytoplasmic inclusions in hepatocytes by light microscopy, which reacted positively for fibrinogen, and a characteristic fingerprint-like appearance of the stored fibrinogen by Electron Microscopy (EM) [1,2,3].

Among the reported FSD cases, two children with the Aguadilla mutation presented with hypofibrinogenemia and hypo-apo-β (APOB)-lipoproteinemia [10,11]. In one of the two cases [11], the molecular analysis of the *APOB* and *Microsomal Triglyceride Transporter Protein* (*MTTP*) genes, which are responsible for lipid transfer and assembly, revealed no abnormalities, thus the low plasma levels of APOB lipoproteins remained unexplained.

Recently, we came across an additional child with the Aguadilla mutation presenting with hypofibrinogenemia associated with hypo-APOB-lipoproteinemia. The recurrence of this association urged us to set up an extensive investigation on this novel Aguadilla case and a retrospective study on the available material from all FSD cases from our files, collected during the period 1987–2017 and including four additional Aguadilla, 1 Ankara, and 1 Brescia cases.

The combination of histology, immunohistochemistry, EM, molecular analysis, and 3-D protein structure studies clarified the pathogenetic mechanism of this intriguing association.

## 2. Results

### 2.1. Proband

#### 2.1.1. Morphological Studies

**Figure 1:**
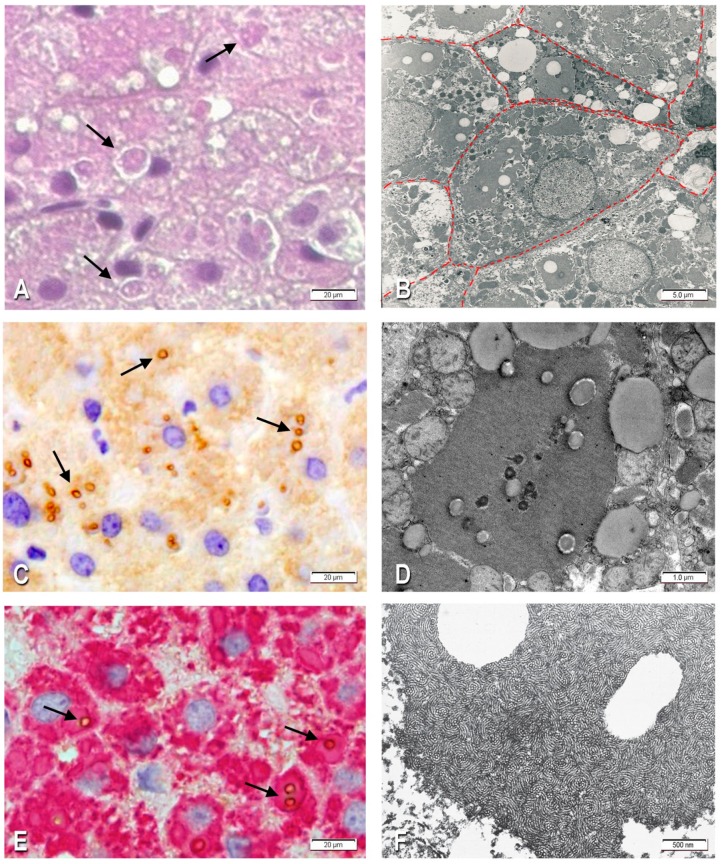
Liver tissue sections from the proband Aguadilla case (**A**) Hematoxilin and Eosin (HE) 100×; the microphotograph shows hepatocytes with plenty of round eosinophilic cytoplasmic inclusions surrounded by a clear halo. Individual inclusions contain empty vacuoles that can be single or multiple (arrows); (**B**) EM 2500×; the electronmicrophotograph from the same case shows four hepatocytes with dilated cisternae of the ER filled up with tubular curved bundles corresponding to fibrinogen. Lipid droplets are present within the fibrinogen. The cytosol contains lipid inclusions of variable size (microsteatosis). A color line draws the hepatocytes shapes; (**C**) Immunostaining of a serial section from the same case stained with an anti-APOB antibody, 100×, showing positivity in the form of single or multiple inclusions. Positivity is strong in the periphery of the droplets (arrows), whilst the central core remains negative; (**D**) EM 12,000×; a large dilated cisterna of ER containing fingerprint-like tubular structures filling up the entire lumen. A few small lipid droplets are located within the tubular structures; (**E**) Double immunostaining of a serial section from the same case, using an anti-fibrinogen and an anti-APOB antibodies sequentially (1000×). Fibrinogen inclusions fill up the entire hepatocyte cytoplasm and are stained in red; the lipid droplets containing APOB are stained in dark brown. The latter can be single or multiple, and the positivity is located at the periphery of the droplets (arrows); (**F**) EM 18,000×; fibrinogen Brescia case. The electronmicrophotograph shows a high magnification of a dilated cisterna of ER filled up with fingerprint-like tubular structures arranged in curved bundles. The fibrinogen inclusion contains two large lipid droplets.

The histological examination of liver tissue sections revealed a preserved lobular architecture with a mild expansion of portal tracts and an occasional portal–portal septum. A mild lymphocytic infiltration was present in a minority of portal tracts without spillover into the parenchyma. Nearly 100% of the hepatocytes contained round or polygonal eosinophilic inclusions filling up the entire cytoplasm (Figure 1A). At times, the inclusions were surrounded by a clear halo and often centered by lipid vacuoles that could be single or in clusters (Figure 1A). Both fibrinogen globules and intraglobular lipids were negative on Periodic Acid Schiff reaction (PAS) or PAS after diastase digestion (PAS-D) staining. A mild degree of microvescicular steatosis was present in several hepatocytes.

#### 2.1.2. Immunohistochemistry

All eosinophilic cytoplasmic inclusions stained positively for fibrinogen and were negative for AAT and α-1-antichymotrypsin, thus indicating a selective and exclusive retention of fibrinogen [2]. The staining for IgG was negative, thus excluding the possibility of a passive inflow of plasma proteins [12]. The intraglobular lipid vacuoles were positively stained with an APOB specific antibody. The positivity was quite strong and localized at the periphery of the vacuoles (Figure 1C). In double immunostained preparations, the APOB immunoreactive material could be visualized within individual fibrinogen-positive inclusions (Figure 1E).

Sections from patients with no mutations in the *FGG* (i.e., without fibrinogen inclusions) were negative when stained with the two antibodies. Likewise, the sections from the case series remained equally negative when the primary antibodies against the two proteins were omitted.

#### 2.1.3. Electron Microscopy

Under the EM, the fibrinogen globular inclusions corresponded to dilated ER cisternae filled up with densely packed tubular structures arranged in curved bundles, resulting in a fingerprint-like appearance (Figure 1B). A majority of fibrinogen globules contained single or multiple lipid inclusions (Figure 1B,D). Nonmembrane-bound lipid droplets were observed within the cytoplasm.

#### 2.1.4. Molecular Genetics Analysis

Sequencing analysis of the *FGA*, *FGB*, *APOB*, *MTTP* genes coding regions and splice sites did not reveal mutations; *FGG* analysis showed the p.R375W (Aguadilla mutation) in a heterozygous status. The genotype of other study cases is reported in Table 1.

#### 2.1.5. Structural Analysis

**Figure 2:**
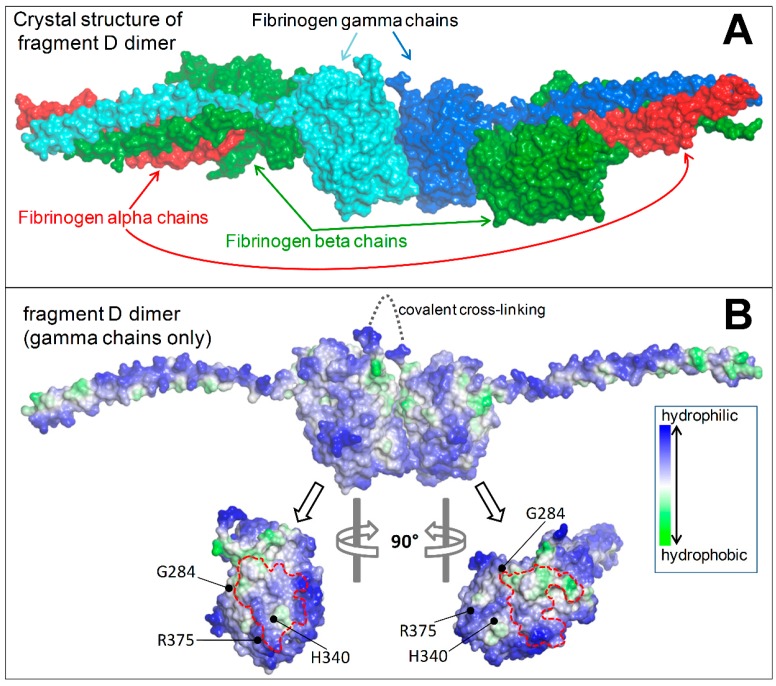
Crystal structure of the covalently bound dimer of fragment D (double-D). (**A**) Molecular surface representation of the fibrinogen chains composing the fragment D dimer (PDB 1FZB); (**B**) The two fibrinogen gamma chains arranged head to head as in the fragment D dimer and after rotations of 90° are shown to highlight the regions of mutual interaction (areas enclosed by the red, dotted lines). Molecular surfaces are colored according to the residue hydrophobicity. The positions of the residues mutated in Fibrinogen Ankara (H340), Aguadilla (R375), and Brescia (G284) are indicated. The covalent link between the gamma chains is schematized by the gray, dotted line.

The analysis of the crystal structure of human fibrinogen fragment D (PDB 1FZB) showed that the Aguadilla, Ankara, and Brescia mutations fall nearby the regions exploited by the globular domains of two γ chains in their “end-to-end” interaction necessary for the D dimer formation, and also that these regions feature patches of hydrophobic residues that normally remain hidden upon correct dimerization (Figure 2).

**Figure 3:**
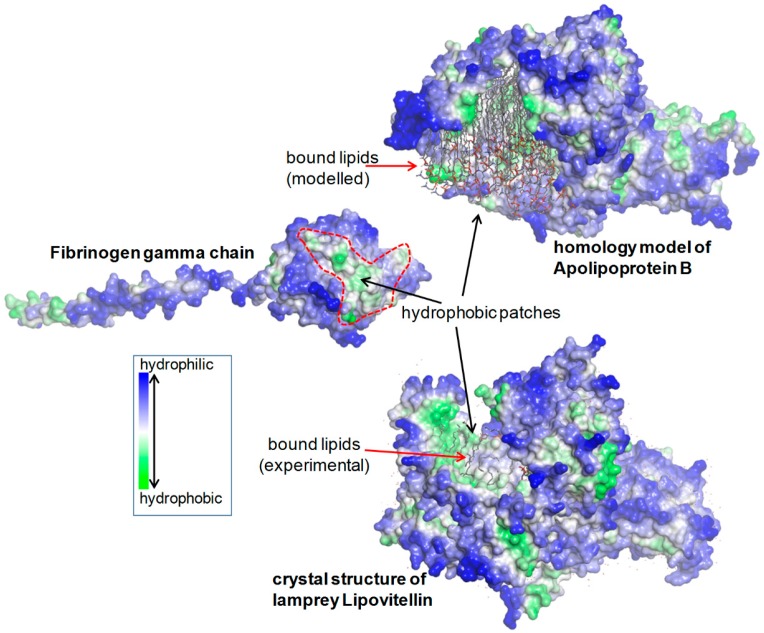
Exposed hydrophobic region in the unpolymerized fibrinogen gamma chain as a potential site for abnormal interactions with APOB. A fibrinogen γ monomer (PDB 1FZB) is shown together with the homology model of APOB with bound lipids [13] and the crystal structure of the closest structurally characterized homologue of the latter, lipovitellin (PDB 1LSH). Molecular surfaces are colored according to the residue hydrophobicity. The partial lipid structures bound to lipovitellin are present in the crystal structure. A failure in fibrinogen polymerization leaves exposed a large hydrophobic region in the gamma chains, which might give rise to undue interactions with lipids and with the hydrophobic regions of APOB and other proteins.

Patches of hydrophobic residues are also present in the lipid-binding region of APOB, as it can be observed by examining the homology model of APOB by Richardson et al. [13] and also in the crystal structure of lipovitellin (PDB 1LSH), which is the closest homologue of APOB with a known folding (Figure 3).

#### 2.1.6. Retrospective Analysis

The review of histological and ultrathin sections from the six previously reported FSD cases led to the identification of lipid droplets within the fibrinogen inclusions at both light microscopy and EM levels. Lipid inclusions were not mentioned in previous FSD case reports, except in the Fibrinogen Brescia case [1,3]. We reviewed the original ultrathin sections from this case and confirmed the presence of lipid inclusions within the aggregated fibrinogen in the ER (Figure 1F).

The main clinical and biochemical results from the study series are summarized in Table 1.

Table 2 summarizes all confirmed cases of FSD in the literature.

## 3. Discussion

This study demonstrated that in FSD due to Aguadilla, Ankara, and Brescia mutations the mutant fibrinogen is retained within the ER in the form of round or polygonal eosinophilic PAS-D-negative inclusions which contain in their inside single or multiple lipid droplets. The lipid droplets correspond to APOB lipoprotein and other lipids. The colocalization of the two components in the same inclusion bodies was confirmed by a double immunostaining technique with the sequential application of an anti-fibrinogen and an anti-APOB lipoprotein antibody on the same liver tissue section. The positivity for APOB was mainly located at the circumferential periphery of the lipid inclusions, whose central empty part corresponded to lipids which were dissolved by solvent agents during the preparation processes. Under the EM, the lipid droplets appeared entrapped within the net of curved fingerprint-like tubular structures corresponding to fibrinogen that aggregated within the ER.

The first point to bring into the discussion is the molecular profile of the regulatory genes of APOB lipoproteins.

Interestingly, the molecular genetic analysis of the two genes, *APOB* and *MTTP,* which are involved in the transfer of triglycerides, assembly, and transport of APOB-containing lipoproteins, failed to demonstrate any mutation in these genes in our FSD cases. These results led to the conclusion that the hypo-APOB-lipoproteinemia associated with hypofibrinogenemia does not fit into the spectrum of hereditary a-betalipoproteinemia (OMIM# 200100). The latter is an autosomal recessive disorder characterized by the virtual absence of VLDL and LDL from plasma [20]. In this disorder, the failure of secretion of VLDL points to defects in the processing of the APOB proteins or to an impairment of the assembly or secretion of triglyceride-rich lipoproteins. The molecular defect was identified in the absence of activity of MTTP, a factor critical to lipidation of APOB.

In familial hypo-beta-lipoproteinemia (FHBL) (OMIM# 107730), a genetic heterogeneous autosomal codominant disorder [21], defects of *APOB* genes are involved in most cases, leading to the formation of prematurely truncated APOB species. However, a number of defects affecting the rate of synthesis or the rate of removal of APOB is also emerging [21].

The second point to bring into discussion is the colocalization of the two proteins (the abnormal fibrinogen and the normal APOB) in the same ER inclusions. This finding is in congruence with the low plasma levels of both proteins.

The third point raises the question about the primary event in the storage process.

The mutations affecting Aguadilla, Ankara, and Brescia are located in the globular domain of the gamma chain, nearby the region of the “end-to-end” interaction that involves two of these molecules in the formation of the D dimer. Mutations located on the D–D interaction surface are capable of resulting in a defective polymerization of fibrinogen [22].

The molecular genetics results from our study definitely ruled out the possibility that APOB lipoproteinemia in FSD was due to genetic reasons, thus we focused on the possible involvement of defective fibrinogen gamma chains in APOB deficiency. As a matter of fact, the globular domain of the fibrinogen γ chain contains patches of hydrophobic residues that are exploited in the formation of the D dimer and therefore remain hidden upon correct polymerization. The γ chain mutations are expected to induce important changes at the site of D–D interactions. This can affect the D–D dimerization, causing the hydrophobic patches to remain abnormally exposed towards the external milieu and available for undue interactions with other molecules in the ER lumen. With regard to the presence of lipid droplets within the fibrinogen inclusions, we can assume that the overexposed hydrophobic patches of particular γ chain mutations can bind APOB molecules, since the latter protein is characterized by large hydrophobic regions that are normally used for the transport of lipids. These hydrophobic features are highlighted in Figure 2, showing the arrangement of fibrinogen α, β, and γ chains and the D–D interface of two gamma chains in the fragment D from the PDB structure 1FZB [22], and in Figure 3 showing a fibrinogen gamma monomer from the previous structure, the homology model of APOB [13], and the crystal structure of the lamprey lipovitellin (PDB 1LSH) [23] that is the closest homologue of APOB with known folding.

γ chain mutants anomalously bound to APOB may serve as nucleation centers for the formation of hydrophobic clusters. Above a critical concentration, the latter coalesce into a lipid phase that becomes visible in the form of lipid droplets at both light and ultrastructural levels.

The reason why the lipid droplets appear empty under the light microscope is that most lipids (triglycerides, cholesterol, LDL cholesterol) disappear as a consequence of the detergent effect of the solvents used during tissue preparation. The APOB protein content, however, is preserved and can be visualized by immunohistochemistry by a specific antigen–antibody reaction. This mechanism explains our morphological observations in congruence with the known biochemical pathways. In fact, during the aggregation process of mutated fibrinogen molecules within the ER, lipid molecules transverse the ER membranes and translocate from the cytosol to the luminal cisternae together with other lipid components. Once there, they are sequestered by the abnormal monomeric fibrinogen gamma chains, which undergo aggregation.

The assembly of lipoproteins containing APOB particles in our FSD cases would have no reasons to be hampered, as the assembly and secretion processes are physiologically regulated by microsomal-associated luminal lipid droplets [24], and these are regularly transferred from the cytosol to the ER when the responsible *APOB* and *MTTP* genes are normal.

The mutant gamma chains are entirely retained as monomers in the ER [25] and never exported into the circulation in the Aguadilla [5], as well as Brescia mutations [3,24].

A fourth point for discussion is the link at the cellular level between basal fibrinogen expression and lipid metabolism. This represents a basic phenomenon in biology [26]. Indeed, it has been demonstrated that the increase of APOB runs parallel to the increased basal expression of fibrinogen and that the overexpression of fibrinogen is associated with increased APOB retention due to a diminished intracellular degradation [26].

In FSD, the low plasma levels of APOB can be sufficiently explained by APOB retention within the secretory pathway. The degradation of both APOB and misfolded fibrinogens follows the ubiquitination proteasome pathway [27,28], which requires dislocation and retrotranslocation of the aberrant proteins towards the cytoplasm with the cooperation of endoplasmic reticulum-associated degradation (ERAD) proteins. ERAD are found in association with cytoplasmic lipid degradation complexes [23,29]. In addition to the ERAD pathway, the excess of aggregated fibrinogen mutants is degraded via autophagy [16].

Recently, it was demonstrated that autophagy-enhancing drugs such as carbamazepine diminish hepatocellular death in both Aguadilla and Brescia FSD [16]. A normalization of transaminases was reported during a long-term follow-up in an Aguadilla child treated with ursodeoxycolic acid [30]. An incomplete response to both carbamazepine and ursodeoxycholic was more recently reported by Mei-Hong Zhang in a Chinese boy with a de novo Aguadilla mutation [19].

Considering that APOB and other lipids are unavoidably retained within the ER in FSD, one would expect that drugs or molecular interventions capable of reducing the amount of fibrinogen aggregation and storage would also result in a decrease of the amount of APOB and other lipids retention and consequently in their plasma level increase.

This study has definitely proven that hereditary hypofibrinogenemia is due to fibrinogen gamma chain mutations and that the associated hypo-APOB-liproteinemia is a secondary phenomenon.

Fibrinogen mutations can provoke conformational changes in the region of the globular domain involved in the “end-to-end” interaction, thus impairing the D dimer formation. Each monomeric fibrinogen gamma chain is left with an abnormal exposure of hydrophobic patches that become available for interaction with APOB, causing intracellular retention and impairment of export.

The phenomenon results in unique morphological features consisting in lipid droplets within fibrinogen globules.

## 4. Material and Methods

The material of the study comprised a prospective patient (proband) (case n. 1, Table 1 and Table 3) and six retrospective cases from our files (cases n. 2–7, Table 1), which were suffering from hereditary hypofibrinogenemia and hepatic storage. All of them had already been published individually by us (see below, Retrospective studies).

### 4.1. Case History (Proband)

A three-year-old female from Turkey, second born to nonconsanguineous parents by vaginal delivery at term. The parents and the brother were in good health. The birth weight was 2750 g. At age 5 months, the child was hospitalized for fever, and abnormal liver function tests were detected: AST 77 U/L (n.v. 20–60), ALT 151 I/U (n.v. 5–45), GGT 67 U/L (n.v. 5–32). During a two years follow-up, the liver test remained abnormal. At the last follow-up physical examination, the patient presented hepatomegaly (3 cm below the right costal margin) and abnormal transaminases (AST 165 U/L, ALT 246 UL, GGT 71 U/L). The child also showed persistent low plasma fibrinogen levels (74 mg/dL; n.v. 200–400) and low levels of APOB-lipoprotein (<24.5 mg/dL; n.v. 55–135), triglycerides (32.6 mg/dL; n.v. 34–112), cholesterol (69.3 mg/dL; n.v. 112–200), and LDL-cholesterol (14 mg/dL; n.v. 63–129). PT, PTT, INR were normal. Because of the persistence of the biochemical alterations, a liver biopsy was obtained. Blood from the child and other members of the family was collected, after informed consent, for molecular analysis. The main biochemical parameters from the child and other family members are summarized in Table 3.

### 4.2. Histology and Immunohistochemistry

Part of the liver specimen was fixed in 10% formalin and embedded in paraffin. Four-micron-thick sections were stained with H.E., PAS, PAS after diastase (PAS-D), and Masson’s trichrome.

Additional serial sections were stained by using the following antibodies against: fibrinogen (1:5000, polyclonal, Dako, Glostrup Municipality, Denmark), AAT (1:1000, ab9399, Abcam, Hong Kong), α-1-antichymotrypsin (ready to use, Dako), IgG (ready to use, Dako), APOB (1:1000, ab7616,).

Double immunostaining for APOB and fibrinogen was carried out on a single tissue section.

As controls, liver tissue sections from the Aguadilla patient were stained by omitting the primary antibodies. In addition, liver tissue sections from patients without hypofibrinogenemia or hypo-APOB-lipoproteinemia were processed and stained by using both primary antibodies.

### 4.3. Electron Microscopy

A small portion of the needle biopsy was fixed in 2.5% glutaraldheyde and embedded in Epon. Semithin sections were cut with a Leica Ultracut S ultramicrotome and stained with toluidine blue. Ultrathin sections were mounted on copper grids, stained with lead citrate and uranyl acetate, and analyzed with a Zeiss Leo 7000 transmission EM (Zeiss, Oberkochen, Germany).

### 4.4. Molecular Genetics Analysis

DNA was extracted, after informed consent, from the peripheral blood (QIAmp DNA Mini kit, Qiagen, Hilden, Germany). The mutation screening was done using polymerase chain reaction (PCR) amplification and DNA sequencing of coding exons and of all splice junctions of *Fibrinogen α chain* (*FGA*, NM_000508), *Fibrinogen β chain* (*FGB*, NM_005141), *Fibrinogen γ chain* (*FGG*, NM_000509), *Apolipoprotein B* (*APOB*, NM_000384) and *Microsomal Triglyceride Transporter Protein* (*MTTP*; NM_000253) genes.

### 4.5. Structural Analysis

The crystal structure with Protein Data Bank, PDB, code 1FZB was used for the molecular structure analysis of the covalently bound dimer of fragment D. The crystal structure of lipovitellin (PDB 1LSH) [23], that is the closest structurally characterized homologue of APOB, was shown together with the fibrinogen γ monomer (PDB 1FZB). The homology model of APOB was obtained from Richardson et al. [13]. The molecular rendering was made with Accelrys Discovery Studio. The hydrophobicity coloring was based on the hydrophobicity scale by Kyte and Doolittle.

### 4.6. Retrospective Studies

Histology, immunohistochemistry, and EM studies were redone in all FSD cases from the files, four cases carrying the Aguadilla mutation [10,11,14,17], one the Ankara [9], and one the Brescia mutation [3]. The study methodology was analogous to that of the proband. The available biochemical data from the case series are summarized in Table A. All cases were characterized by low plasma fibrinogen levels, and four Aguadilla cases and the Ankara case had concomitant hypo-APOB-lipoproteinemia. The levels of APOB lipoprotein were not available in the remaining two cases (one Aguadilla and one Brescia).

## 5. Conclusions

In conclusion, this study has demonstrated that a scrupulous examination of routinely stained histological sections can allow the recognition of the elementary lesions underlying this intriguing phenomenon. Immunohistochemistry, EM, molecular genetics analysis, and 3-D protein structure analysis have contributed in confirming the light and electron microscopic findings and in unraveling the pathogenetic mechanisms.

It would be interesting to check whether the phenomenon occurs also with other FSD mutations.

Our report is expected to encourage pathologists to continue to report new cases of FSD and clinicians to design appropriate trials aimed to reduce the liver storage and plasma deficiency of the two proteins.

## Figures and Tables

**Table 1:** Ethnic, molecular genetics, biochemical, and morphological findings in seven fibrinogen storage disease (FSD) cases.

Case	Age	Sex	Ethn	Mutation	Fibrinogen (Normal Value-n.v. 200–400)	APO-B	TChol	LDLChol	Triglyc	Lipid Inclusion	Liver Path
1 Proband	2 y	F	Turkey	Aguadilla	74	<24.5	69.3	14	32.6	+++	Portal and septal fibrosis
2	5 y	F	Turkey	Aguadilla	48	<22.1	69	37	28	++	Mild
3	4 y	M	Italy	Aguadilla	43	NA	NA	NA	NA	++	Septal fibrosis
4	2 y	M	Japan	Aguadilla	37.6	22	76	32	45	+++	Early cirrhosis
5	3 y	F	Saudi A.	Aguadilla	89	NA	NA	NA	NA	+++	Mild
6	5.5 y	F	Turkey	Ankara	49	75	169	99	100	++	Mild
7	49 y	F	Italy	Brescia	20	NA	NA	NA	NA	+++	Cirrhosis

**Table 2:** Literature review for confirmed cases of *FGG* mutations.

Reference	Mutant Fibrinogen	Age (Year)	Gender	Clinical Presentation	Liver Disease
[3]					
Index	Brescia	64	M	Elevated ALT/AST	Severe
6 adult family members			5 F & 1 M		Mild
[5]					
Index	Aguadilla	3	F	Elevated ALT/AST	Mild
Sibling		7	F	Elevated ALT/AST	Mild
Father		NA	M		Mild
[14]					
Index	Aguadilla	6	M	Elevated ALT/AST	Severe
[15]					
Index	Aguadilla	61	M	Elevated ALT/AST	Severe
Son		28	M	Normal ALT/AST	Mild
Son		22	M	Normal ALT/AST	
[6]					
Index	Anger	35	F	Elevated ALT/AST	Severe
3 adult family members			2 F & 1 M		Mild
[11]					
Index	Aguadilla	2	M	Elevated ALT/AST and hypo-β-lipoproteinemia	Moderate–Severe
Father				Normal ALT/AST	
[7]					
Index	Al duPont	4	M	Elevated ALT/AST and mild coagulopathy	Mild
[16]					
Index	Aguadilla	6	F	Elevated ALT/AST	Mild
[16]					
Index	Brescia	5	M	Elevated ALT/AST	Mild
[17]					
Index	Aguadilla	3	F	Elevated ALT/AST	Mild
Father		42			
[10]					
Index	Aguadilla	7	F	Elevated ALT/AST and hypo-β-lipoproteinemia	Mild
Mother		33	F	Normal ALT/AST	
Brother		11	M	Normal ALT/AST	
Sister I		9	F	Normal ALT/AST	
Sister II		5	F	Elevated ALT/AST	
[18]					
Index	Aguadilla	4.6	F	Fibrinogen = 70 mg/dL Elevated ALT/AST	NA
Mother				Elevated ALT/AST	NA
Grandmother				Elevated ALT/AST	NA
[8]					
Index	Aguadilla	3	F	Fibrinogen (immunological 117 (n.v. 160–400)	Normal Liver
Mother					
Maternal grandfather					
[8]					
Index	Aguadilla	3	M	Fibrinogen 66 mg/dL Elevated ALT/AST	Normal Liver
Mother					
Brother					
[9]					
Index	Ankara	5.5	F	Elevated ALT/AST Hypo-APOB-lipoproteinemia	Mild
Father					
[19]					
Index	Aguadilla (de novo)	2	M	Fibrinogen 29mg/dL	Portal fibrosis and mild hepatitis
Present Case					
Index	Aguadilla	3	F	Elevated ALT/AST hypo-APOB-lipoprotereinemia	Mild
Mother		24	F	Normal ALT/AST	NA

**Table 3:** Proband’s family studies: main clinical, biochemical, and molecular genetics results.

	Proband	Sibling	Mother	Father
Age	2 years	4 years	24 years	36 years
Gender	F	M	F	M
Hepatomegaly	-	-	-	-
Splenomegaly	-	-	-	-
AST, U/L (n.v. 20–60)	77	25	15	30
ALT, U/L (n.v. 5–45)	151	13	13	47
GGT, U/L (n.v. 5–32)	67	10	10	35
ALP, U/L (n.v. 145–420)	316	216	83	67
Triglyceride, mg/dL (n.v. 34–112)	32.6	75	39.8	282.6
Cholesterol, mg/dL (n.v. 112–200)	69.3	176.8	167.7	245.7
LDL-Cholesterol, mg/dL (n.v. 63–129)	14	103	95	152
APOB mg/dL (n.v. 55–135)	<24.5	NA	NA	128
PT, s. (n.v. 12.1–14.5)	14.8	NA	NA	11.3
INR (n.v. 0.92–1.14)	1.27	NA	NA	0.95
PTT, s. 1 (n.v. 25–34)	26	NA	NA	25
Fibrinogen, mg/dL (n.v. 200–400)	74	NA	140	292
Liver biopsy	+	NA	NA	NA
Molecular analysis	Aguadilla	-	Aguadilla	-

n.v. = normal value; AST, aspartate transferase; ALT, alanine amino transferase; GGT, gamma glutamil transpeptidase; ALP, alkaline phosphatase; PT, prothrombin time; INR, international normalised ratio; PTT, thrombin partial time; NA, not assessed.

## Aknowledgments

The Author wish to thank Federica Salutari, Marta Stefanelli, and Riccardo Mariani for the skilled technological assistance and Marco Tartaglia for constructive discussion and critical review of the manuscript. We would like to thank Martin K. Jones and Jere Segrest at Vanderbilt University for providing us with the structure file of the homology model of APOB described in their paper: Richardson, P.E., Manchekar, M.; Dashti, N.; Jones, M.K.; Beigneux, A.; Young, S.G.; Harvey, S.C.; Segrest, J.P. “Assembly of lipoprotein particles containing apolipoprotein-B: structural model for the nascent lipoprotein particle”, *Biophys. J.* **2005**, *88*, 2789–2800.
